# Factors Associated with Methadone Treatment Duration: A Cox Regression Analysis

**DOI:** 10.1371/journal.pone.0123687

**Published:** 2015-04-14

**Authors:** Chao-Kuang Lin, Chia-Chun Hung, Ching-Yi Peng, En Chao, Tony Szu-Hsien Lee

**Affiliations:** 1 Education Center for Humanities and Social Sciences, National Yang-Ming University, Taipei, Taiwan; 2 Department of Psychiatry, Taichung Veterans General Hospital, Taichung, Taiwan; 3 Department of Health Promotion and Health Education, National Taiwan Normal University, Taipei, Taiwan; Medical University of Vienna, AUSTRIA

## Abstract

This study examined retention rates and associated predictors of methadone maintenance treatment (MMT) duration among 128 newly admitted patients in Taiwan. A semi-structured questionnaire was used to obtain demographic and drug use history. Daily records of methadone taken and test results for HIV, HCV, and morphine toxicology were taken from a computerized medical registry. Cox regression analyses were performed to examine factors associated with MMT duration. MMT retention rates were 80.5%, 68.8%, 53.9%, and 41.4% for 3, 6, 12, and 18 months, respectively. Excluding 38 patients incarcerated during the study period, retention rates were 81.1%, 73.3%, 61.1%, and 48.9% for 3 months, 6 months, 12 months, and 18 months, respectively. No participant seroconverted to HIV and 1 died during the 18-months follow-up. Results showed that being female, imprisonment, a longer distance from house to clinic, having a lower methadone dose after 30 days, being HCV positive, and in the New Taipei city program predicted early patient dropout. The findings suggest favorable MMT outcomes of HIV seroincidence and mortality. Results indicate that the need to minimize travel distance and to provide programs that meet women’s requirements justify expansion of MMT clinics in Taiwan.

## Introduction

Treating heroin dependence and stemming the human immunodeficiency virus (HIV) infection amongst heroin users challenge public health professionals and health providers over the last three decades. The use of methadone maintenance treatment (MMT) to treat heroin dependence and reduce infectious diseases has gradually become prevalent. Countries in South East Asia and East Asia, including China, Indonesia, Malaysia, Thailand, and Vietnam, have been launching and expanding MMT programs as an essential response to reduce the harms of injection drug use. This trend of adopting harm reduction as an approach to reduce the ill effects of heroin dependency followed a series of important announcements and recommendations made by international organizations. In 2002, the United Nations Office on Drugs and Crime (UNODC) announced that based on systematically reviewed evidence from addiction studies, drug addiction should be considered a chronic and relapsing disease [1]. Later on, other international health organizations, including the World Health Organization (WHO) and the Joint United Nations Program on HIV/AIDS (UNAIDS), recommended harm reduction programs as best practices and crucial for reducing HIV infection among injecting drug users (IDU) [2]. In Taiwan, MMT as a harm reduction program was introduced in 2006 throughout the nation to stem the spread of HIV amongst heroin injection users.

Methadone maintenance treatment (MMT) has been shown to reliably reduce drug use [3], overdose mortality [4], HIV seroconversion [5], and risk behavior [6] in heroin-dependent patients. As staying longer in MMT has been associated with various desirable outcomes [7,8], retention has proven to be of value as a proximal indicator of the effects of MMT. In both prospective and retrospective studies, the incidence of new HIV infections has been found to be significantly reduced with longer duration of MMT [6,9]. Other studies have found that age, gender, employment, duration of heroin use, HIV, treatment cost, methadone dosage, and treatment satisfaction are associated with MMT duration [10–13]. Although MMT is widely acknowledged as effective in reducing heroin dependence and HIV infection in Western societies, its adoption by psychiatrists, methadone patients, and Taiwanese society as a whole has been relatively recent. Given the limited clinical experience in Taiwan, it is imperative to know how long patients had stayed in MMT and which factors associated with treatment dropout in Taiwan. Hence, researching patients’ behavior and clinical characteristics associated with duration in MMT as well as HIV seroconversion can be critical in redesigning and improving the quality of treatment services. In this paper, we report data from an 18-month prospective cohort study on MMT retention rates and factors associated with the duration of MMT in Taiwan.

## Methods

### Design

This paper reports secondary analyses from a larger project entitled “A Prospective Study of Effectiveness of Methadone Maintenance Treatment,” an 18-month multi-site study that collected data on quality of life, mortality and HIV seroconversion as well as on the duration of MMT and associated factors [14]. The research protocol was reviewed and approved by the Ethics Committee of Human Subjects Protection at Taipei Medical University and Taipei City Hospital in Taiwan. A total of 599 participants who met the criteria in the Diagnostic and Statistical Manual of Mental Disorders (DSM-IV-TR) were recruited consecutively by the psychiatrists in 4 outpatient MMT sites in northern Taiwan between July 2008 and April 2010. After being informed about the purpose and procedures of the study, patients signed consent forms, including consent for the researchers to contact the Ministry of Justice to find out if they are incarcerated. MMT began with a dosage at 30mg/day and was individually titrated as needed. All patients were reassessed monthly to adjust the dosage based on the standard MMT protocol in Taiwan. Patients had to go every day to the clinic to receive the methadone doses and were considered “treatment dropouts” if they failed to take their methadone for 14 consecutive days.

### Participants

Patients had to meet the following criteria to be included in the study: at least 20 years of age, literate, self-reported usage of heroin within the past 6 months, and currently enrolled in an MMT program. In accordance with the Taiwan Civic Law, being 20 years old is considered as an adult. Of the 599 participants, we analyzed 128 newly admitted patients, who were approached with an explanation of the study’s purpose and procedures within 5 days of starting MMT. The participants were followed semi-annually for 18 months. All participants received 100 Taiwan dollars (approximately 3 US dollars) for each interview.

### Assessments

A semi-structured questionnaire was used to obtain demographic and drug use history. The demographic items asked about the participant’s age, age at first heroin use, gender, education, living address, marital status, and employment. Education was categorized based on whether the patients had completed the compulsory 9 years of education in Taiwan. House-to-clinic distance was calculated from the patient’s home address to the MMT clinic using a Google map. Results of HIV and HCV tests, daily records of methadone taken, and morphine toxicology test results were taken from a computerized medical registry. Mortality data were obtained from the death records of the Taiwan Department of Health for the corresponding period. Arrest and incarceration records were provided by the Taiwan Ministry of Justice.

### Statistical analyses

Statistical analyses were performed using SPSS 19.0 for Windows. MMT duration was calculated from records of patients’ admission and dropout dates or the last day of follow-up (April 30, 2010). Cox multivariate regression analyses were performed twice, once on all 128 patients and once on non-incarcerated patients, to predict treatment dropout from age, age at first heroin use, gender, education, house-to-clinic distance, marital status, employment, incarceration, methadone dose after 30 days, and program site. Items with missing values were omitted from the analyses. The criterion for statistical significance was p < 0.05.

## Results

As shown in Table 1, participants’ mean age was 36.65(8.34) years and the mean age at first heroin use was 27.25(7.07) years; 88.3% were male, 73.4% had at least nine years of education, 66.4% were employed, 53.9% were single and 26.6% married, 9.4% were HIV positive, and 89.8% were HCV positive. On average, the daily dosages of methadone after 30 days of admission were 61.27(26.31) mg.

**Table 1:** Background information on 128 newly admitted participants and hazard ratios from a Cox multivariate regression analysis on treatment dropout within the 18-month prospective follow-up period.

Variable	Mean(S.D.)	HR	95% CI of HR	
			Lower	Upper
Retention duration (days)	344.65(203.68)			
Age	36.65(8.34)	1.010	0.982	1.039
Age at first heroin use	27.25(7.07)	0.993	0.948	1.040
House-to-clinic distance (km)	5.63(5.01)	1.083**	1.035	1.133
Methadone dose after 30 days (mg)	61.27(26.31)	0.989**	0.983	0.996
	N (%)			
Incarcerated during study period				
No	90(70.3)	0.438**	0.251	0.763
Yes	38(29.7)	Referent		
Gender				
Male	113(88.3)	0.445*	0.207	0.957
Female	15(11.7)	Referent		
Education				
Less than 9 years	34(26.6)	1.049	0.612	1.798
At least 9 years	94(73.4)	Referent			
Employed				
Yes	85(66.4)	0.76	0.402	1.439
No	43(33.6)	Referent		
Marital status				
Single	69(53.9)	1.285	0.501	3.301
Divorced/Widowed	25(19.5)	1.794	0.893	3.601
Married	34(26.6)	Referent		
HIV serostatus				
Positive	12(9.4)	0.554	0.252	1.220
Negative	116(90.6)	Referent		
HCV serostatus				
Positive	115(89.8)	3.914*	1.013	15.119
Negative	13(10.2)	Referent		
Program site				
New Taipei city	36(28.1)	2.838*	1.063	7.578
Keelung city	62(48.4)	0.589	0.228	1.522
Yilan city	19(14.8)	2.888	0.956	8.728
Taipei city	11(8.6)	Referent		

Note. HR = hazard ratio. CI = confidence interval.

*p<.05.

**p <.01.

The average number of days participants were in MMT was 344.65(203.68). Four patients withdrew from MMT within 30 days of treatment. Retention rates were 80.5% (n = 103), 68.8% (n = 88), 53.9% (n = 69), and 41.4% (n = 53) for individuals who consistently stayed in treatment for 3 months, 6 months, 12 months, and 18 months respectively. Of 128 participants, 38(29.7%) had to leave MMT during the study because they had been convicted of a crime and became incarcerated. For the remaining 90 patients, retention rates were 81.1% (n = 73), 73.3% (n = 66), 61.1% (n = 55), and 48.9% (n = 44) for those who stayed in treatment consistently for 3 months, 6 months, 12 months, and 18 months, respectively. None were seroconverted to HIV positive and one died because of accidents unrelated to overdose. The annual mortality rate was 0.5% per life year.

The Cox regression analysis on the 128 patients revealed that patients who were women, imprisoned, HCV positive, lived a long distance from the clinic, low methadone dose after 30 days of admission, and treated in New Taipei City were associated with shorter duration of MMT (Table 1). The Cox regression performed on the 90 patients not incarcerated during the follow-up period indicated that being female, HCV positive, living a longer distance from the clinic, and receiving treatment in New Taipei City, respectively, were associated with shorter durations of MMT.

## Discussion

We found that being male, married, living close to the clinic, living in Taipei City, and being prescribed a higher methadone dosage 30 days after admission, HCV positive and not imprisoned were positively associated with continuing (not dropping out of) MMT. No patient was tested HIV positive and one died because of accidents unrelated to overdose during the study period. These findings are consistent with prior studies showing that staying longer in MMT is associated with such positive patient outcomes as lower overdose mortality, fewer HIV/HCV infections, less drug use overall, fewer injections, and a better quality of life [4,15].

Studies from Western countries have shown that younger patients are the most likely to drop out of substance abuse treatment programs [16] and that females stay in longer than males [17]. However, women in this study had shorter duration in MMT. A previous study examining gender differences in 1,892 heroin users receiving MMT in Taiwan found that women were more likely to have family members using illicit substances, begin MMT earlier after initial heroin use and report child bearing burden [18]. Another earlier study on 270 female heroin users found that most of them experienced violence and sexual assault by male drug users [19]. We speculated that females in treatment dropped out due to the reasons that they were embarrassed or worried about male acquaintances or no one can take care of their young child.

One third of our participants became incarcerated during the 18months of the study, and incarceration was a significant predictor of early attrition. A previous study found involvement in the criminal justice system to be associated with less time in treatment [20]. Legal problems, defined by composite scores on the ASI, were also found to be related to poorer MMT retention [11]. Many of the MMT patients in our sample were incarcerated because they were caught using illegal drugs. In Taiwan, once patients are detained by the criminal justice system, they are not able to resume their MMT treatment. That is to say, incarcerated MMT patients must stop both their illegal drug use and their methadone regimen “cold turkey” the day they enter the correction facility. Dolan et al. [21] found that heroin users who received MMT during incarceration had reduced mortality rates, re-incarceration rates, and hepatitis infections. These results reinforce our recommendation that MMT be integrated into prison settings to ensure optimal recovery outcomes.

Higher methadone doses have also been associated with longer duration in MMT [16,22]. In a recent nationwide study in Taiwan, Liao et al. [23] found that not only does an adequate dosage of methadone ease the cravings and withdrawal symptoms of MMT patients, but it also reduces their mortality rate. In addition, a higher MMT dosage has been related to a better quality of life [15]. Although our results replicate previous findings that a higher dose is associated with higher retention, even six months after admission the MMT patients we studied were generally receiving much less than the minimum methadone doses recommended by Villafranca et al. [16]. It is interesting that once the incarcerated participants were removed from the study sample, low methadone dose became insignificant. This finding may relate to the fact that many study patients were incarcerated because they were caught using illegal drugs. We also speculate that the significant finding about early treatment dropout is probably attributable to the MMT provider factors in the New Taipei City such as methadone personnel trait, staff deployment, or time slots of giving methadone. Further research is needed to verify whether treatment providers can be a significant factor in treatment retention.

Our finding that distance from the clinic is associated with MMT retention is noteworthy but not surprising, because travel costs are important considerations for patients needing access to treatment. Because methadone is a 24-hour agonist that patients must take every day, they may have to take a half day off from work or spend more money on transportation to keep up with their medication. In such circumstances, reporting daily to the MMT clinic can be a heavy burden in terms of both time and cost for patients who live far from the clinic. Accessibility to clinics has been shown to be a crucial factor influencing MMT adherence and retention [10,13]. Kelly et al. [11] found that convenience of the clinic is a critical indicator of treatment satisfaction for MMT patients, which in turn is a significant predictor of 12- month treatment retention. In Taiwan, public transportation is often lacking for patients who live in suburban areas. The distance from home to clinic is a serious concern for such patients, particularly if they do not own a motor vehicle. Thus, our finding suggests that the network of methadone clinics in Taiwan should be expanded to improve accessibility through adjustments in the logistics of program operation. These adjustments include extending clinic hours, offering transportation services, and providing take-home doses if feasible.

### Limitations

The present study has a few limitations. As our participants were not recruited randomly from methadone clinics in northern Taiwan, the findings may not generalize to other Taiwanese methadone patients. Although our clinic-based sample was large, our participants still may have differed from the general population of heroin users in relevant respects. Examples of such possible differences are demographics, MMT modality, and patterns of drug use. Second, because ours was an observational study with no comparison group, confounding factors such as maturation, local policy change, and testing may have biased our results. The average number of days in treatment was fairly long in our study. Previous studies suggest that patients who remain in MMT programs more than three months are much more likely to stay in the program longer than other patients [11,24]. The final limitation of our study is that we did not include provider factors, which can influence treatment retention, treatment quality, and provision of ancillary services or counseling.

## Conclusion

An important strength of this study lies in its ability to use a prospective design. As a result, the conclusions of this study can provide evidence that MMT as a harm reduction program reduce the mortality and new HIV infections. Factors associated with duration of MMT are gender, methadone dose after 30 days, incarceration, program site, HCV and the geographic distance that clients travel to outpatient treatment clinics. Minimizing the travel distance might improve retention and duration of MMT.
